# Developing and validating a rapid assessment tool for small ruminant reproduction and production in pastoralist flocks in Kajiado, Kenya

**DOI:** 10.1016/j.vas.2021.100186

**Published:** 2021-06-17

**Authors:** Cristina Ballesteros, Amos Mwasi, Eunice Mungai, Awo Ibarahim, Christine Thuranina-McKeever, Gabriel Oluga Aboge, Joshua Orungo Onono, Pablo Alarcon

**Affiliations:** a Veterinary Epidemiology, Economics and Public Health, Department of Pathobiology and Population Sciences, The Royal Veterinary College, Hawkshead Lane, North Mymms, Hatfield, Herts, AL9 7TA, United Kingdom; b Department of Public Health, Pharmacology and Toxicology, University of Nairobi, P.O. BOX 29053, 00625 Kangemi, Nairobi, Kenya; c London Centre for Integrative Research on Agriculture and Health, London, United Kingdom

**Keywords:** Pastoralist, Small ruminant, Reproduction, Performance, Efficiency, Indicator

## Abstract

The collection of production performance data in small ruminants pastoralists flocks is essential to evaluate their efficiency and assess how different challenges, such as diseases, droughts or changes in land use, affect their sustainability. Although different methodologies exist, capturing these data is difficult, particularly in nomadic pastoral flocks. In this study, a method for rapid assessment of flock production performance based on farmer recall was designed, implemented and tested.

Through literature review and consultation with pastoralists and key informants, a questionnaire was developed to capture small ruminant flock data disaggregated by age (≤2 years old and >2 years old), species and sex. Data on flock dynamics and on reproduction parameters were collected for a period of 12 months. A survey of 130 pastoralists with medium to large flocks was then conducted in Kajiado County (Kenya). Values were calculated for 12 reproduction performance indicators and 7 additional production performance indicators. In addition, a flock efficiency indicator (FEI) is proposed that classifies mixed flocks (i.e., with sheep and goats) into low, medium or high efficiency.

Results showed that in flocks with low efficiency, the median value for net fecundity rate was 0.43 lambs/ewe (range 0.08–1.00) and 0.41 kids/doe (range 0.07–0.73), and the median production rate was 6% (range [−47%] to 20%) for sheep and 11% (range [−38%] to 0.21%) for goats. In flocks with high efficiency the median net fecundity rate was 0.77 lambs/ewe (range 0.48–2.73) and 0.88 kids/doe (range 0.49–1.80), and the median production rate was 25% (range 11–47) for sheep and 28% (range 15–46) for goats. Sixty-two (47.7%) of the pastoralists surveyed reported usually buying animals into their flocks, and, consequently were considered as ‘pastoralists and traders’. Their flocks had significantly lower FEI scores (median = 7.5, interquartile range [IQR] 6–9), compared with “pastoralists only” flocks (median = 8, IQR 6–10, Kruskal–Wallis rank sum test, *p*-value <0.001).

Since this method is based on pastoralist recall, values obtained should be considered as approximations. Nonetheless, the proposed assessment tool can be used by individuals with low resources or recording capacity, and in large scale programmes to monitor pastoralist flock dynamics, set-up benchmarking programmes, estimate the impact of diseases and shocks, identify those flocks which are most vulnerable to these shocks and evaluate the effectiveness of policies and interventions on herd performance.

## Highlights

• A methodology to capture data on pastoralists’ flock performance was designed, implemented and tested.
• Data on 18 productive parameters for sheep and 19 for goats from 130 pastoralist flocks were obtained.
• A flock efficiency indicator, combining four of the parameters obtained is suggested for rapid evaluation of the flocks.
• Poorer herd performance was found amongst those pastoralists that also reported trader activity.

## 1 Introduction

Pastoralist-based livestock systems are an essential livelihood strategy in arid and semi-arid lands that are characterized by soils with poor fertility and too dry for crop production (GoK, 2013; ILRI, 2019; Nyariki & Amwata, 2019). They are also a source of prestige and wealth, conferring a cultural identity onto these rural communities (GoK, 2012; Nyariki & Amwata, 2019). The importance of small ruminants in these areas is widely recognized; they are the main species kept by pastoralists because they are able to survive the harsh climate and environment (Abassa, 1995; Ojango, Oyieng, Audho & Okeyo Mwai, 2014). Their production is common in vulnerable households in pastoral settings, providing a source of food and economic security (Abassa, 1995; Bekure, de Leeuw, Grandin & Neate, 1991).

In 2015, Kenya's small ruminant population was estimated at 17 million sheep and 25.8 million goats. About 57% of sheep and 50% of goats raised in Kenya are kept in the arid and semi-arid lands, which are predominantly inhabited by pastoralists (Nyariki & Amwata, 2019). It is estimated that around 80% of all meat consumed in the country is from pastoralist systems (Nyariki & Amwata, 2019). Moreover, it is predicted that the demand for mutton and goat meat in Kenya will increase by 46% in 2050 (FAO, 2017). Kajiado County, with a population of 1,120,000 sheep and 877,744 goats in 2019 (Knoema, 2020), is the seventh largest producer of sheep and the eleventh largest producer of goats in Kenya.

Despite their importance, improving production in pastoralist systems is limited by numerous challenges, such as those posed by climatic shocks (e.g. droughts), prevalence of infectious diseases and increased pressure on natural resources, particularly the progressive shift to private land ownership (FAO, 2017; GoK, 2013; Homewood, Kristjanson & Trench, 2009). To mitigate these challenges, it is first essential that the efficiency (production and economic performance) of these systems is measured and monitored. Improvements to the production system can then be implemented to support their sustainability. Additionally, the effectiveness of interventions and policies within this sector can be evaluated. Yet, there is no system currently in place to regularly measure the productivity of small ruminant flocks owned by pastoralists. Existing methods are implemented in very few flocks, normally breeding nucleus flocks or those involved in research programmes. The absence of monitoring systems and the low productivity of flocks have been highlighted as the main challenges facing pastoralists in Kenya (GoK, 2013). Reasons for this lack of data include the difficulty of accessing pastoralists, the complexity of capturing production and economic data, and consequently, an absence of record keeping.

Flock production performance parameters can be divided into reproductive performance indicators (those related to the reproduction of the animals) and other production parameters associated with the survival, trade and growth of animals. Reproduction performance indicators (RPIs) may include ‘proportion of adult ewes/does that lamb/kid’, ‘parturition interval’, ‘proportion of lambs/kids born alive or dead’, ‘litter size’ and ‘age at first lambing/kidding’, as well as occurrence of reproduction problems such as ‘abortion’, ‘dystocia’, ‘ewe/doe mortality’ and ‘malformation’. RPIs are important because reproduction failure is the first sign of severely-constrained resources (Abassa, 1995). Other production parameters are related to flock dynamics such as *‘*mortality rate’, ‘offtake rate’ and ‘intake rate’. Different approaches have been implemented for collecting production performance data for small ruminant flocks owned by pastoralists: animal-based flock follow-up, flock follow-up and retrospective survey based on farmer recall of flock demography (Lesnoff, Messad & Juanès, 2016; Marshall, Ejlertsen & Poole, 2011). However, these data collection systems are highly labor and time intensive, since inputs and outputs are normally collected from individual animals in longitudinal studies or through the completion of long questionnaires. Hence, these methods are often expensive and not practical for sustainable large-scale implementation in Kenya or other low and middle-income country (LMIC) settings. Rapid tools that can capture and measure production performance indicators are therefore needed.

Currently, without reliable and up-to-date data, detection and management of pastoralists’ production inefficiencies and resources is difficult, and often requires the use of subjective views from point sources. This lack of data acts as a barrier for pastoralists to improve their flocks’ production and manage their resources efficiently; and for government services to provide advice or effectively implement and monitor interventions or policies. Furthermore, if RPIs are significantly impacted, this risks the ability of pastoralists to maintain their flock size, threatening their food security and livelihood. The objective of this study was to develop a tool for rapid assessment of the production performance indicators of small ruminants raised in medium and large flocks in pastoralist areas and assess its feasibility. This tool will provide support for pastoralists, extension officers and policy makers when making decisions that aim to improve small ruminant production in LMICs. Moreover, the baseline information on production performance indicators generated here can be used for future research and evaluation of small ruminants in other pastoralist areas.

## 2 Materials and methods

### 2.1 Study area

This was a cross-sectional survey involving small ruminant pastoralist flocks in Kajiado County, Kenya. This county was selected because pastoralism is the main source of livelihood and because of the accessibility of the area to researchers (GoK, 2013). The county is located in the southern part of Kenya and covers an area of 21,900 km^2^. There are five administrative sub-counties, namely: Kajiado North, Kajiado Central, Kajiado East, Kajiado West and Kajiado South. These are further sub-divided into 25 wards.

Kajiado County is considered an area modestly vulnerable to climate change (vulnerability index of 0.426) (MoALF, 2017). It is predominantly semi-arid and characterized by extreme temperatures and cyclical droughts. Its rainfall pattern is bi-modal, with a short rainy season between October and December and a long rainy season between March and May. However, this pattern has become increasingly unpredictable, with damaging consequences on people's livelihoods. As an example, the drought that occurred in 2009 is estimated to have caused 70% of livestock losses and 90% of crop failures (GoK, 2013). Furthermore, land tenure and use in Kajiado County is undergoing significant change, resulting in increased competition for resources (Homewood et al., 2009).

**Fig. 1:**
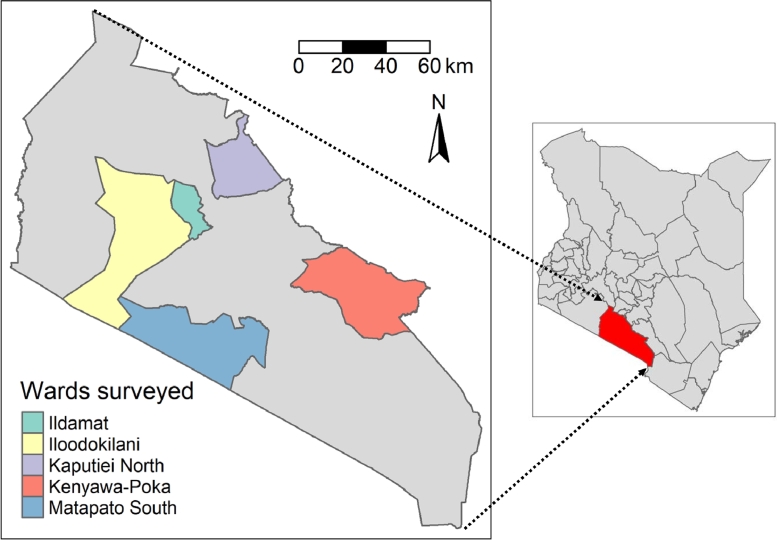
Kenya map highlighting Kajiado wards included in the study.

Two wards from Kajiado East (Kaputiei North and Kenyawa-Poka), two wards from Kajiado Central (Ildamat and Matapato South) and one ward from Kajiado West (Iloodokilani) were selected for this study based on livestock densities and accessibility following the advice of the Directorate of Veterinary Services (DVS) of Kajiado County (Fig. 1).

### 2.2 Selection of study participants

Pastoralist flocks were selected with the active support of staff from the DVS, who supported introduction to pastoralist communities. The study focused on “medium and large size” flocks. These were defined as flocks with more than 10 ewes and/or more than 10 does, and a minimum overall flock size of 30 small ruminants of at least one species. Thus, the study included “reproductive flocks”, in other words flocks containing enough females to be able to renew by themselves.

A sample size of 130 flocks was estimated to generate accurate baseline data for reproduction and production performance indicators from small ruminants at farm level. This sample size allowed for the determination of the true mean of each indicator (being this a continuous variable) in the sheep and goat (separately) flock population with 95% confidence interval, assuming a standard deviation of 10 (e.g. abortion rate as continuous variable).

Completely random sampling of small ruminant flocks was not possible due to a lack of accessibility in some areas. Flocks were systematically sampled based on transect drives along rural feeder roads within each of the selected wards. Initially the aim was to sample flocks evenly across all five wards (26 in each). From each manyatta (group of Maasai households sharing close family ties and relationships and keeping their cattle herds, sheep and goat flocks together during grazing) situated along the transects, only one flock was selected and included in the study. However, when a selected pastoralist household declined to participate in the study, a replacement was sought from the same manyatta. When replacement was not possible from within a ward then additional households were sampled from the other wards.

### 2.3 Data collection

Following informed consent, face-to-face interviews were conducted with the flock owner and/or the herdsman using a structured questionnaire. The questionnaire was developed following interviews with several pastoralists, expert consultation in Kenya and literature review (Lesnoff, 2008, 2011; Lesnoff et al., 2016; Otte & Chilonda, 2002).The questionnaire was piloted with 7 pastoralists in Kajiado County to test its acceptability, clarity and efficiency in capturing responses. To minimize bias when collecting data, enumerators were trained for a period of four weeks on how to use tablets for data capture and transmission, and how to approach respondents in the field. In addition, they were involved in piloting data collection tools.

Data were collected on flock size and structure; and retrospective data on reproduction, mortality, offtake and intake for a period of 12 months. Data on mortality were categorised as: “diseases”, “drought”, “predation” and “other reasons”; offtake data were categorised as: “sale”, “slaughter for own consumption”, “gift/dowry/inheritance”, “loss” and “other reasons”; finally intake data were categorised as: “purchase”, gift/dowry/inheritance” and “other reasons”. The inclusion of these categories allowed us to characterize and quantify the main reason(s) for entry/exit of animals in the flock and also enhanced the recall capacity for the respondents.

Data were collected separately for sheep and goats, and four categories were used to collect animal data for flock entries and exits as well as current flock structure: “young females” (ewes or does younger than or equal to 2 years old); “young males” (rams or bucks younger than or equal to 2 years old); “reproductive females” (ewes or does older than 2 years old) and “older males” (rams or bucks older than 2 years old). Although the reproductive age of animals was variable, pastoralists involved in the study identified the age of 2 years in both sheep and goats as the best cut-off to differentiate between reproductive and non-reproductive or immature animals. Reliability for this cut-off was tested during piloting of the questionnaire. When a pastoralist owned more than one flock, data were collected for all the flocks. Other characteristics of interest, such as main breed(s) present in the flock, adverse events suffered by the flock during the previous 12 month period and replacement strategy for new animals were also collected during the interview. A version of the questionnaire with the relevant questions is available in Annex 1.

All interviews were conducted in an eight-week period between June and August 2018 to ensure that data collected from different flocks referred to a similar retrospective period. The questionnaire was administered using the Open Data Kit (ODK) software (https://opendatakit.org/). This enabled the collection of data in electronic format on tablets in the field, which minimized data entry errors. The questionnaire was conducted in Kiswahili. When another language was required, DVS staff present was able to support translation.

### 2.4 Data analysis

Different performance indicators such as parturition, prolificacy, mortality, offtake and intake rates and others, were calculated. In total, 19 production parameters were estimated (Table 1) and the formulas to calculate them are shown in Annex 2. In summary, the number of reproductive females present on the date of the survey, as proposed by Lesnoff (2009), was used as the denominator for calculating different reproductive parameters. To calculate mortality, intake, offtake and production rates, the number of animal-years at risk was used as the denominator. This was calculated using an approximate denominator approach based on the arithmetic mean between flock sizes at the beginning and the end of the study period (FAO, 2018; Lessnoff, 2015). Flock size at the beginning of the 12 month period was obtained by subtracting entries and adding exits to the number of animals reported in the flock at the date of interview.

**Table 1:** Production parameters estimated in the present survey Definitions of parameters were taken and adapted from Lesnoff (2016) and Marshall (2011).

Parameter definition	
Parturition rate	Average number of parturitions per reproductive female^a^over a year
Prolificacy rate	Average number of offspring (stillborn or born alive) per parturition
Twinning rate	Proportion of parturitions that were twins
Triplet rate	Proportion of parturitions that were triplets
Fecundity rate	Average number of offspring (both stillborn and born alive are included) per reproductive female per year
Abortion rate	Annual hazard rate of abortion for a reproductive female
Stillbirth rate	Probability that an offspring is a stillborn over a year
Dystocia rate	Probability of a difficult birth over a year
Net prolificacy rate	Average number of offspring born alive per parturition
Net fecundity rate	Average of offspring born alive per reproductive female
Average age at first parturition	
Reported age of the reproductive female at replacement	
Multiplication rate	Calculated as annual multiplication rate. Flock size at date of survey/ Flock size 12 months before the date of survey.
Growth rate	Calculated as percentage annual growth rate. 100 × (annual multiplication rate - 1)
Production rate	Calculated as annual production rate.*P*/*N*, where:*P* = (flock size at date of survey - flock size 12 months before the survey) + (number of offtakes over the year - number of intakes over the year),and *N* is the mean flock size over the year. Note that *P* represents the balance between births and deaths
Net production rate	Calculated as annual net production rate. Balance between offtakes and intakes over the mean flock size during the year.
Mortality rate	Annual hazard rate of natural death (natural death refers to all types of death except slaughtering: diseases, predation, drought and any other cause of death).
Intake rate	Annual hazard rate of enter the flock as intake (purchases, gift/dowry/inheritance and “other reasons”)
Offtake rate	Annual hazard rate of exit the flock as offtake. It includes slaughtering for own consumption, sales, gift/dowry/inheritance, lost animals and “other reasons”

^a^Reproductive females were defined by females older than two years.

Summary statistics of the parameters obtained were calculated and the results were compared with existing values from available literature. Shapiro–Wilk test was used to test for Normality of all the indicators. As data were not normally distributed, the median and range were reported. An exploratory analysis of outliers was performed for values exceeding the lower bound, equal to the 25th percentile minus [1.5 x interquartile range (IQR)], and the upper bound, equal to the 75th percentile plus (1.5 x IQR) (Tukey, 1977). A Spearman's correlation analysis was conducted to investigate the dependencies among the different indicators. A correlation matrix was then displayed to allow a further characterization of the parameters and the presence of collinearity.

Different analytical approaches were considered to allow a further characterization of the flocks. In the first instance, a principal component analysis (PCA) was explored for identification of potential components that summarize different performance indicators. Subsequently, a cluster analysis using a K-means clustering approach was performed, using a selection of performance indicators, to seek subsets of flocks with similar characteristics. The indicators selected were sheep net fecundity rate, goat net fecundity rate, sheep flock multiplication rate, goat flock multiplication rate, sheep net production rate and goat net production rate. Those variables were selected because: (i) these represent a more reliable estimate and were obtained with fewer questions; (ii) these are parameters providing an indication of overall reproduction and flock performance; and (iii) they are indicators related to flock sustainability.

Finally, a new production parameter was developed, the Flock Efficiency Indicator (FEI), based on two indicators for both species. Net fecundity rate was selected because it provides an indication of overall reproduction performance, while the production rate provides an indicator of post-birth production performance. A combination of both performance indicators can be used to determine the overall performance of the flock. For this, each variable was categorized in a three-point efficiency score (1 for low efficiency and 3 for high efficiency), with cut-off points based on each variable's tertiles. The flock performance indicator was then calculated as the sum of all four variables:

$$\begin{array}{c} Flock\;Efficicency\;\;Indicator\\ =Sheep\;Net\;Fecundity_{\;cat\;}+Goat\;Net\;Fecundity_{cat\;}\\ +Sheep\;Production\;rate_{cat}+\;Goat\;Production\;rate_{cat\;} \end{array}$$

Where *cat* refers to the efficiency score described above. Multiplication rate was not considered for this indicator, as it is based on intake and offtakes, which may not be related to flock performance, but instead to the consequences of good or poor flock performance (i.e., poor performance may prompt pastoralists to purchase a larger number of animals, and hence increase their multiplication rate).

The FEI was then compared with other flock indicators. In particular a Kruskal–Wallis test was used to assess the associations between the FEI and the multiplication and net production rates in sheep and goats separately to further characterize pastoralists. Furthermore, difference in overall efficiency between those who reported usually buying animals to bring into their flocks, considered as “pastoralists and traders” and those who did not buy animals regularly, classified as “pastoralists only”, was investigated. Microsoft Excel (Microsoft Corporation, Redmond, WA, USA) and R Version 3.6.2 (The R Foundation for Statistical Computing) were used for data manipulation and analysis.

## 3 Results

### 3.1 Description of flocks sampled

A total of 134 small ruminant pastoralists were interviewed. However, interviews with four pastoralists failed to capture the essential data needed and were discarded from the analysis. The questionnaire took an average of 45 min to be completed, but questions relating to production performance were estimated to take 25 min (estimation based on feedback from interviewers).

In total, data were obtained from 130 flocks: 27 from Ildamat and 25 from Matapato South, located in Kajiado central; 31 from Kaputiei North and 21 from Kenyawa–Poka, located in Kajiado East; and 26 from Iloodokilani, located in Kajado West. One flock did not have any sheep and 15 did not have any goats. In addition, two respondents reported that they were in the process of removing goats from their flocks in order to solely raise sheep. Regarding the breed composition of flocks, cross-bred sheep were the most prevalent in 69 flocks (53.5%) and only in few flocks did they coexist with purebred animals (3 flocks included Red Maasai and 3 flocks included Dorper sheep). In 50 (38.8%) flocks where Dorper sheep was the most prevalent type of breed, 19 included cross-breeds and another 9 flocks included Red Maasai. In 56 (48.7%) flocks where cross-bred goats was the most prevalent type of breed reported, 4 included Galla sheep; and in 56 flocks where Galla goats was the most prevalent breed, 19 flocks also included cross-breeds. A complete description of the baseline characteristics of flocks surveyed is given in Table 2 and Table 3.

**Table 2:** Baseline characteristics of the flocks surveyed (Total number of flocks = 130).

Variable	*n* (%)
Usually buy animals to bring into the flock	62 (47.7)
The flock is not located in a manyatta	127 (97.7)
Main reason for keeping sheep and goats	
Regular cash income	124 (95.4)
Insurance against emergencies	4 (3.1)
Prestige	1 (0.8)
Milk production	1 (0.8)
Number of flocks owned	
1	120 (92.3)
2	7(5.4)
3	2 (1.5)
4	1 (0.8)
Main breed of sheep present in the flock(s)^a^ ***n*** ***=*** ***129*** (1 flock without sheep)	
Crosses	69 (53.5)
Dorper	50 (38.8)
Red maasai	10 (7.8)
Main breed of goat present in the flock(s)^a^ ***n*** ***=*** ***115*** (15 flocks without goats)	
Crosses	56 (48.7)
Galla	56 (48.7)
Small East Africa	3 (2.6)
Prevention of unwanted breeding in sheep ***n*** ***=*** ***129*** (1 flock without sheep)	88 (68.2)
Prevention of unwanted breeding in goats ***n*** ***=*** ***115*** (15 flocks without goats)	71 (61.7)
Flock suffered an adverse event during the previous year	66 (50.8)
Disease	32 (24.6)
Disease and predation	17 (13.1)
Disease and drought	9 (6.9)
Disease and theft	3 (2.3)
Drought	2 (1.5)
Drought and predation	1 (0.8)
Disease, drought and predation	1 (0.8)
Disease, drought and heat stress	1 (0.8)

^a^Main breed is reported although different breeds and crosses co-exist in some of the flocks.

**Table 3:** Baseline characteristics of the flocks surveyed (Total number of flocks = 130).

Variable	Median	Range
Number of small ruminants owned by the pastoralist	80	30–500
Number of sheep present in the flock(s)	55	7–300
Proportion of young ewes ≤ 2 years	0.22	0–0.57
Proportion of young rams ≤ 2 years	0.17	0–0.36
Proportion of ewes > 2 years	0.49	0.18–0.76
Proportion of rams > 2 years	0.11	0–0.35
Number of goats present in the flock(s)	35	3–241
Proportion of young does ≤ 2 years	0.21	0.05–0.50
Proportion of young bucks ≤ 2 years	0.17	0–0.35
Proportion of does > 2 years	0.50	0.20–0.78
Proportion of bucks > 2 years	0.11	0–0.50

### 3.2 Summary statistic results of production parameters

Those flocks with less than 10 sheep or less than 10 goats, and those that reported that they were purposely removing goats from the flock, were excluded from the analysis, when appropriate, to calculate performance parameters. Thus, 128 flocks were included in the calculation of the parameters for sheep and 111 flocks were included for goats. Tables 4,5 provide median (and range) values obtained for the parameters calculated.

**Table 4:** Productive parameters in sheep^a^(Total number of flocks = 128).

	Md (Mn−Mx)	Mean (se)	Values reported in the literature	References
**Parturition rate**	0.54 (0.08−2.73)	0.61 (0.03)	0.83	Diawara et al., 2017
			0.82	Ejlertsen et al., 2012a
			0.74	Ejlertsen et al., 2012b
			0.65	Marshall et al., 2011
			90.0–106.9	Otte & Chilonda, 2002
**Prolificacy or litter size**	1.04 (1.00−1.71)	1.09 (0.01)	1.04 1.00 1.09 1.00–1.27	Diawara et al., 2017 Ejlertsen et al., 2012a [Bibr bib0020][Bibr bib0024]
**Twinning rate**	0.04 (0.00−0.71)	0.09 (0.01)	0.04	Abassa, 1995
**Fecundity rate**	0.57 (0.08−2.72)	0.66 (0.04)	1.42 0.87	Ejlertsen et al., 2012a, Marshall et al., 2011
**Abortion rate**	0.00 (0.00−0.23)	0.03 (0.00)	0.03 0.11 0.08 0.08	Diawara et al., 2017, Ejlertsen et al., 2012a, Ejlertsen et al., 2012b, Marshall et al., 2011
**Stillbirth rate**	0.00 (0.00−0.40)	0.02 (0.00)	0.07 0.01	Ejlertsen et al., 2012a, Marshall et al., 2011
**Dystocia rate**	0.03 (0.00−1.00)	0.13 (0.02)	NA	NA
**Net prolificacy rate**	1.00 (0.75−1.71)	1.07 (0.01)	1.05 1.19	Ejlertsen et al., 2012a, Marshall et al., 2011
**Net fecundity rate**	0.57 (0.08−2.73)	0.64 (0.03)	1.32 1.33	Ejlertsen et al., 2012a, Marshall et al., 2011
**Reported age at first parturition (years)**	2 (1−4)	2.12 (0.09)	1–3 years 18.8 months	Marshall et al., 2011, Otte & Chilonda, 2002
**Reported age of ewe at replacement**	7 (4−10)	6.93 (0.13)	Not reported	–
**Multiplication rate**	1.03 (0.29−4.00)	1.00 (0.03)	1.06	Marshall et al., 2011
**Growth rate (%)**	3.49 ((−70.59)−300)	0.33 (3.23)	6% 5.7%	Marshall et al., 2011, Otte & Chilonda, 2002
**Production rate**	0.14 ((−0.47)−0.93)	0.14 (0.01)	(21,9 ± 23,9) 0.12	[Bibr bib0004][Bibr bib0020]
**Net production rate**	0.12 ((−0.32)−1.00)	0.10 (0.02)	(−5 ± 29,3) 0.02	[Bibr bib0004][Bibr bib0020]
**Mortality rate**	0.10 (0.00−0.58)	0.14 (0.01)	0.27 0.06 0.32	Ejlertsen et al., 2012a, Ejlertsen et al., 2012b; Marshall et al., 2011
***By mortality event:***				
- **Disease**	0.07 (0.00−0.47)	0.09 (0.01)	Not reported	–
- **Predation**	0.00 (0.00−0.27)	0.04 (0.01)	Not reported	–
- **Drought**	0.00 (0.00−0.32)	0.01 (0.00)	Not reported	–
- **Other causes**	Not reported	Not reported	Not reported	–
**Intake rate**	0.01 (0.00−1.04)	0.08 (0.01)	0.10 0.03 0.07	Ejlertsen et al., 2012a, Ejlertsen et al., 2012b; Marshall et al., 2011
***By intake event:***				
- **Purchase**	0.00 (0.00−1.04)	0.08 (0.01)	0.09 0.03 0.06	Ejlertsen et al., 2012a, Ejlertsen et al., 2012b; Marshall et al., 2011
- **Gift/dowry/inheritance**	0.00 (0.00−0.22)	0.01 (0.00)	0.01 0 0.00	Ejlertsen et al., 2012a, Ejlertsen et al., 2012b; Marshall et al., 2011
- **Other reasons**	0.00 (0.00−0.07)	0.00 (0.00)	Not reported	–
**Offtake rate**	0.19 (0.00−0.91)	0.27 (0.02)	0.34 0.20 0.15 0.19–0.25	Ejlertsen et al., 2012a Ejlertsen et al., 2012b [Bibr bib0020][Bibr bib0024]
***By offtake event:***				
- **Sale**	0.13 (0.00−0.91)	0.18 (0.02)	0.15 0.14 0.06	Ejlertsen et al., 2012ª Ejlertsen et al., 2012b; Marshall et al., 2011
- **Slaughter for own consumption**	0.02 (0.00−0.27)	0.04 (0.00)	0.13 0.03 0.04	Ejlertsen et al., 2012a, Ejlertsen et al., 2012b; Marshall et al., 2011
- **Gift/dowry/inheritance**	0.00 (0.00−0.21)	0.02 (0.00)	0 0 0.02	Ejlertsen et al., 2012a, Ejlertsen et al., 2012b; Marshall et al., 2011
- **Lost**	0.00 (0.00−0.31)	0.03 (0.01)	Not reported	–
- **Other reasons**	0.00 (0.00−0.09)	0.00 (0.00)	Not reported	–

^a^Values reported for 2018, year-to-year variations should be expected.

Abbreviations: Md: median; Mn: Minimum; Mx: Maximum; se: standard error.

**Table 5:** Productive parameters in goats^a^(Total number of flocks = 111).

	Md (Mn−Mx)	Mean (se)	Values reported in the literature	References
**Parturition rate**	0.51(0.07−2.00)	0.61 (0.03)	0.93 0.99 0.75 0.68 0.80–1.40	[Bibr bib0004][Bibr bib0005][Bibr bib0006][Bibr bib0020] Otte & Chilonda, 2002
**Prolificacy or litter size**	1.12(1.00−3.00)	1.2(0.03)	1.14 1.50 1.28 1.10–1.34	Diawara et al., 2017, Ejlertsen et al., 2012a; Marshall et al., 2011; Otte & Chilonda, 2002
**Twinning rate**	0.11 (0.00−0.26)	0.20 (0.03)	0.14	Abassa, 1995
**Triplet rate**	0.00 (0.00−0.20)	0.00 (0.00)	0.14	Abassa, 1995
**Fecundity rate**	0.61 (0.07−2.33)	0.61 (0.04)	1.49 1.10	Ejlertsen et al., 2012a, Marshall et al., 2011
**Abortion rate**	0.00 (0.00−0.63)	0.04 (0.01)	0.19 0.12 0.08 0.11 0.06	Diawara et al., 2017, Ejlertsen et al., 2012a; Ejlertsen et al., 2012b; Marshall et al., 2011 Warui et al., 2007
**Stillbirth rate**	0.00 (0.00−0.00)	0.02 (0.00)	0.06	Ejlertsen et al., 2012a, Marshall et al., 2011
**Dystocia rate**	0.08 (0.00−1.00)	0.16 (0.02)	NA	NA
**Net prolificacy rate**	1.09 (0.90−3.00)	1.18 (0.03)	1.41 1.19	Ejlertsen et al., 2012a, Marshall et al., 2011
**Net fecundity rate**	0.59 (0.07−2.33)	0.68 (0.04)	1.40 0.82 1.43	Ejlertsen et al., 2012a, Ejlertsen et al., 2012b, Marshall et al., 2011
**Reported age at first parturition (years)**	2 (1−4)	2.45 (0.09)	1–3 years 16.6 months	Marshall et al., 2011, Otte & Chilonda, 2002
**Reported age of doe at replacement**	7 (2−10)	6.95 (0.23)	Not reported	–
**Multiplication rate**	1.04 (0.21−2.10)	1.03 (0.03)	1.10	Marshall et al., 2011
**Growth rate (%)**	4.35 ((−79.09)−110)	2.93 (2.59)	10% 4.4%	Marshall et al., 2011, Otte & Chilonda, 2002
**Production rate**	0.20 ((−0.38)−0.58)	0.18 (0.01)	(20,2 ± 22,9) 0.22	[Bibr bib0004][Bibr bib0020]
**Net production rate**	0.15 (0.03−1.29)	0.18 (0.02)	(5,7 ± 31,2) 0.14	[Bibr bib0004][Bibr bib0020]
**Mortality rate**	0.07 (0.00−0.67)	0.12 (0.01)	0.16 0.06 0.34	Ejlertsen et al., 2012a, Ejlertsen et al., 2012b; Marshall et al., 2011
***By mortality event:***				
- **Disease**	0.06 (0.00−0.67)	0.10 (0.01)	Not reported	–
- **Predation**	0.00 (0.00−0.21)	0.02 (0.00)	Not reported	–
- **Drought**	0.00 (0.00−0.41)	0.01 (0.00)	Not reported	–
- **Other causes**	Not reported	Not reported	Not reported	–
**Intake rate**	0.03 (0.00−0.72)	0.07 (0.01)	0.04 0.03 0.06	Ejlertsen et al., 2012a, Ejlertsen et al., 2012b; Marshall et al., 2011
***By intake event:***				
- **Purchase**	0.00 (0.00−0.72)	0.06 (0.01)	0.04 0.03 0.04	Ejlertsen et al., 2012a, Ejlertsen et al., 2012b; Marshall et al., 2011
- **Gift/dowry/inheritance**	0.00 (0.00−0.14)	0.01 (0.00)	0 0 0.00	Ejlertsen et al., 2012a, Ejlertsen et al., 2012b; Marshall et al., 2011
- **Other reasons**	0.00 (0.00−0.06)	0.00 (0.00)	Not reported	–
**Offtake rate**	0.23 (0.00−1.35)	0.25 (0.02)	0.25 0.14 0.21 0.13–0.21	Ejlertsen et al., 2012a, Ejlertsen et al., 2012b; Marshall et al., 2011; Otte & Chilonda, 2002
***By offtake event:***				
- **Sale**	0.11 (0.00−1.20)	0.17 (0.02)	0.13 0.09 0.07	Ejlertsen et al., 2012ª Ejlertsen et al., 2012b; Marshall et al., 2011
- **Slaughter for own consumption**	0.03 (0.00−0.23)	0.04 (0.00)	0.10 0.03 0.07	Ejlertsen et al., 2012a, Ejlertsen et al., 2012b; Marshall et al., 2011
- **Gift/dowry/inheritance**	0.00 (0.00−0.29)	0.03 (0.00)	0.00 0 0.03	Ejlertsen et al., 2012a, Ejlertsen et al., 2012b; Marshall et al., 2011
- **Lost**	0.00 (0.00−0.14)	0.01 (0.00)	Not reported	–
- **Other reasons**	0.00 (0.00−0.14)	0.00 (0.00)	Not reported	–

^a^Values reported for 2018, year-to-year variations should be expected.

Abbreviations: Md: median; Mn: Minimum; Mx: Maximum; se: standard error.

Exploratory analysis showed that only an average of six outliers were found for each parameter calculated, with no or slight impact (variation of less than 3%) on the median values estimated. Most outliers (80%) were found in those parameters indicating reproductive disorders and also in production performance indicators (such as production rate, intake rate, etc.) reported by “pastoralists and traders”. They were included in parameter calculations because their trustworthiness was considered similar to the rest of the values, and the summary statistic (i.e., median) reported is robust and not heavily affected by skewness and outliers.

Average number of offspring born alive per reproductive ewe over the year was 0.57. In goats, the average number of offspring born alive per reproductive doe over the study period was 0.59. Twinning rate was very low for both species and the triplet rate in goats was insignificant. Very few abortions or dystocias were reported over the study period.

Overall mortality rate was 10% in sheep and 7% in goats. The most frequent cause of mortality was reported as disease in both species; 111 (86.7%) in sheep and 78 (70.2%) in goat flocks. On average 74% of mortality reported in sheep and 81% in goats was due to disease and younger animals were the most affected by mortality due to disease. The second most frequent cause of mortality was predation, which was reported by 60 (46.9%) and 27 (24.3%) pastoralists with sheep and goat flocks, respectively. Deaths due to drought were reported by 12 (9.4%) and 10 (9.0%) pastoralists with sheep and goat flocks, respectively. Other causes of mortality were almost negligible; they were reported only by 3 pastoralists with sheep flocks and not reported in goats.

The overall intake rate was very low for sheep (1%) and goats (3%). Sixty-seven (52.3%) and 58 (52.3%) pastoralists reported entry of sheep and goats, respectively, into their flock during the previous 12 months. Almost half of pastoralists reported they had purchased sheep (49.2%) and less than half reported they had purchased goats (43.2%) over the previous year. Animals were mainly purchased in the market. Young ewes (average of 55.7% of the total sheep purchased) and young does (average of 59.2% of the total goat purchased) were the main type of animals purchased in each species. Twenty-nine (22.7%) pastoralists reported they had received sheep as a gift, dowry, or inheritance and 26 (23.4%) reported they had received goats for these reasons. These were mainly young ewes (average of 88.8%) and young does (average of 77.2%). No other reasons were reported for entry of animals into the flocks during the previous 12 months.

The most frequent reason for animals leaving the flock was sale, with most pastoralists selling to the market. Older males were the primary category sold, with pastoralists reporting that they sold on average 63.7% of older rams and 66.5% of older bucks. One average, pastoralists reported selling on average 32.9% of all their ewes and 31.2% of all their does.

Slaughter for own consumption was found to be the second most frequent reason for offtake, with 81 (63.3%) pastoralists reporting that they had slaughtered sheep and 68 (61.3%) that they had slaughtered goats. Most animals slaughtered were older than 2, with older rams representing an average of 67.7% of all sheep slaughtered and older bucks an average of 73.7% of goats slaughtered. “Loss of animal” during the previous 12 months (i.e., animals lost, potentially including stolen animals) was identified as the third most frequent reason for offtake in sheep (in 43 (33.6%) flocks) and the fourth in goats (in 29 (26.1%) flocks). Young females were the main category affected, representing an average of 60.2% of sheep losses and a 46.3% of goat losses. Gifts, dowry or inheritance were reported as a reason for animals exiting in 58 (45.3%) of sheep flocks and 46 (41.4%) of goat flocks. About three quarters of sheep that exited for these reasons were young females, while 12.9% were sheep older than 2 years old. Similarly, on average 68.3% of goats exiting for these reasons were young females, while 15.1% were reproductive does. Male animals from both species were rarely gifted. Other reasons for exit were negligible and reported by only 3 pastoralists (2.3%).

**Fig. 2:**
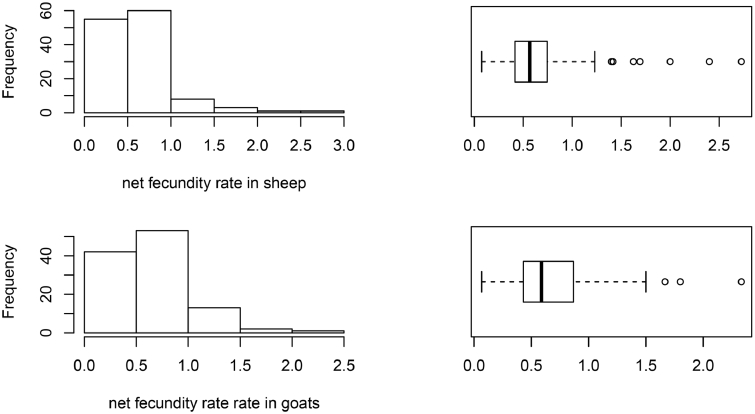

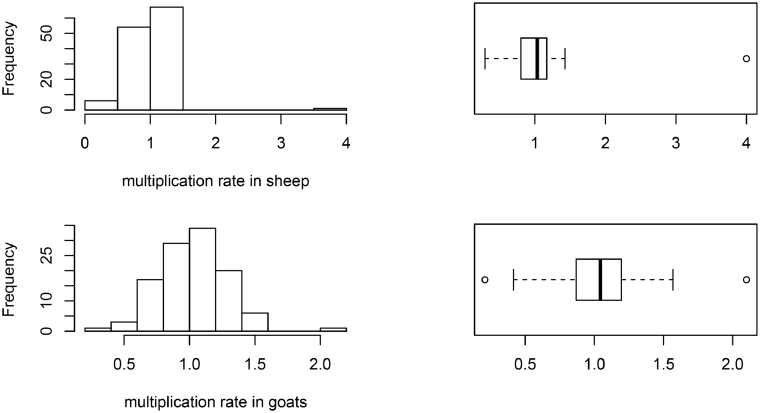

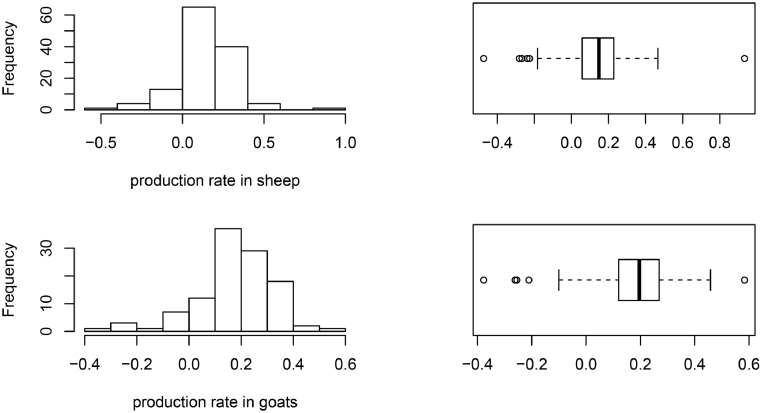
Histogram and boxplot of net fecundity rate (2A), multiplication rate (2B) and production rate (2C) in sheep and goats (Number of flocks: 128 for sheep and 111 for goats).

Overall, the average production rate was 14% in sheep. However, 46% of sheep flocks had reduced in size over the previous 12 months (multiplication factor <1). For goats, the average production rate was 20%, and 40.5% of goat flocks had reduced in size over the previous 12 months. The histograms and box plot of the variables incorporated in the flock performance analysis (net fecundity rate, production rate and fecundity rate) are shown in Fig. 2. They were included in the supplementary information for the rest of the variables (Annex 3).

### 3.3 Correlation analysis of productive parameters

**Fig. 3:**
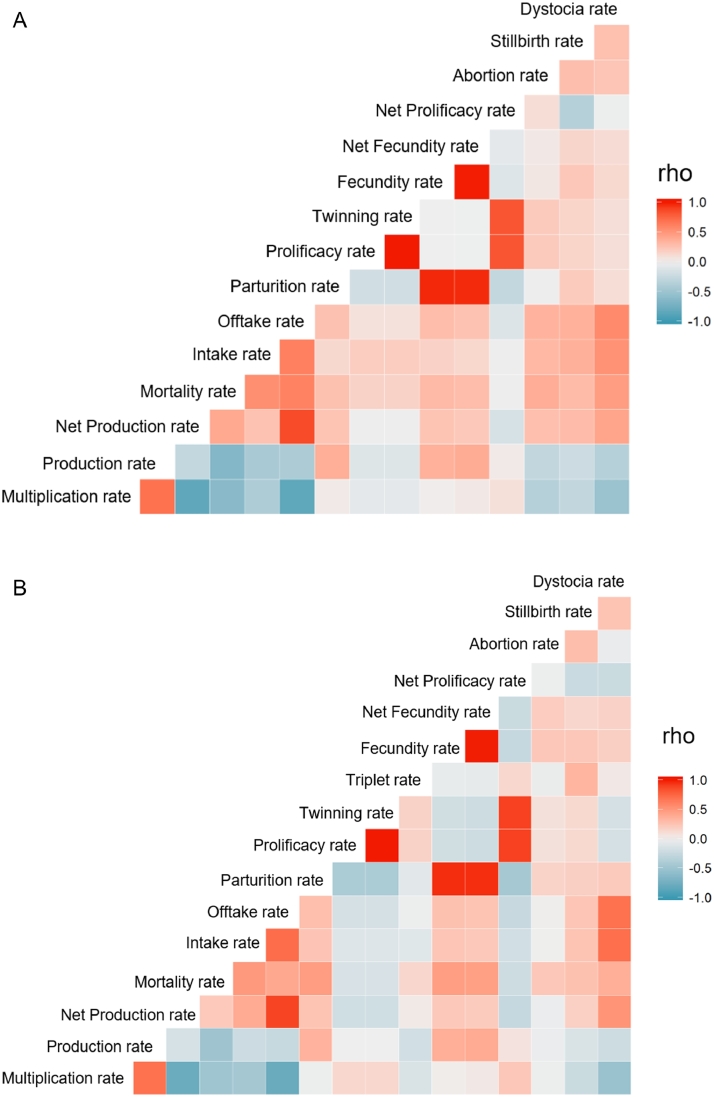
Correlation matrix of the production indicators for sheep (3.a.) and goats (3.b.) (rho indicates Spearman correlation).

The correlation matrix (shown in Fig. 3) revealed that most of the flock performance parameters related to the whole flock was correlated, as expected, as well as the reproductive parameters. Multiplication rate was positively correlated with production rate (*r* = 0.69), *p*-value <0.001 for sheep and goats), but negatively correlated with net production rate (*r* = 0.83, *p*-value <0.001 for sheep and *r* = −0.78, *p*-value <0.001 for goats) and intake rate ( = −0.46, *p*-value <0.001 for sheep and goats).

Fecundity rate, net fecundity rate and parturition rate in sheep were positively correlated with production rate in sheep (*r* = 0.35, *p*-value <0.001; *r* = 0.38, *p*-value <0.001; and *r* = 0.37, *p*-value <0.001, respectively). The same relationship was found in goats (*r* = 0.26, *p*-value <0.001; *r* = 0.39, *p*-value <0.001; and *r* = 0.37, *p*-value <0.001, respectively). Dystocia rate was negatively correlated with multiplication rate (*r* = −0.52, *p*-value <0.001 for sheep and goats) and positively correlated with net production rate (*r* = 0.37, *p*-value <0.001 for sheep and *r* = 0.55, *p*-value <0.001 for goats) and mortality rate (*r* = 0.46, *p*-value <0.001 for sheep and *r* = 0.37, *p*-value <0.001 for goats).

Abortion rates were also positively correlated with mortality rate in both species (*r* = 0.37, p-value <0.001 for sheep; *r* = 0.22, *p*-value = 0.019 for goats). However, abortion rate was only found to be negatively correlated with multiplication rate in sheep (*r* = −0.35, *p*-value <0.001). Stillbirth was also positively correlated with mortality rate (*r* = 0.29, *p*-value = 0.002 for sheep and *r* = 0.26, *p*-value = 0.007 for goats).

The results of the principal component analysis did not provide any meaningful reduction of variables. Similarly, the cluster analysis did not provide distinct or meaningful groups either. The results of both analyses are presented in the annexes 4 and 5.

### 3.4 Results of flock efficiency indicator

**Fig. 4:**
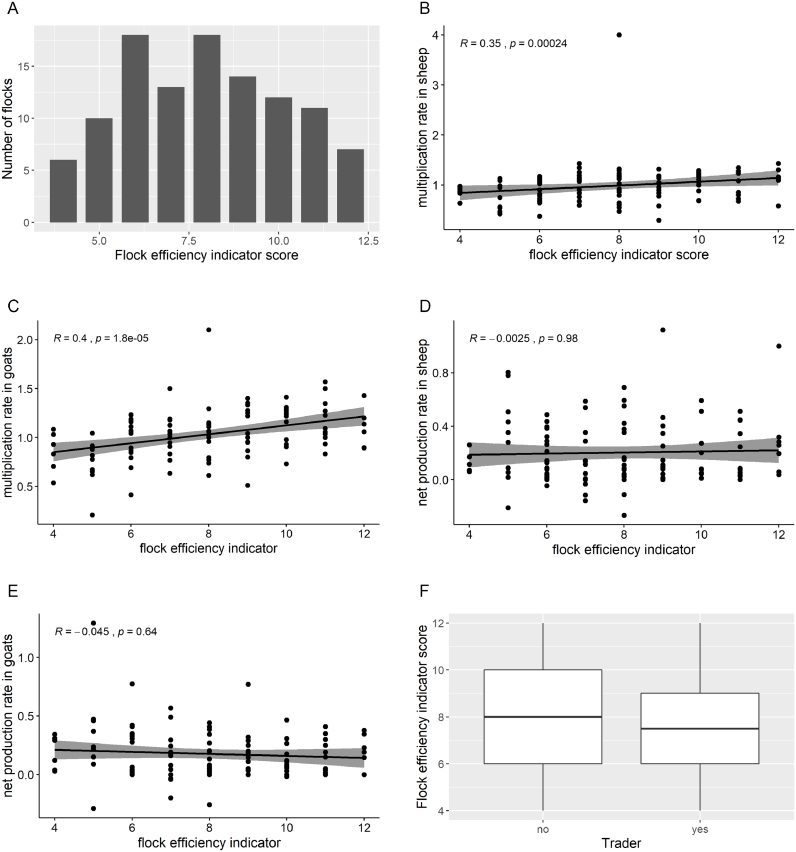
Bar plot of the flock efficiency indicator (4A). Scatter plot of the flock efficiency indicator and multiplication rate in sheep (4B) and goats (4C). Scatter plot of the flock efficiency indicator and the net production rate in sheep (4D) and in goats (4E). Boxplot (4F) comparing the flock efficiency score obtained depending on the type of pastoralist (“pastoralists-only” and “pastoralists-and-traders”).

This new indicator, FEI, was calculated in those 109 flocks with both sheep and goats present (excluding those flocks with less than 30 small ruminants present, less than 10 animals by species or removing goats from the flocks). Fig. 4 shows the descriptive analysis of this new indicator and its correlation with multiplication rate and the net production rate. The median of the FEI was 8 (range 4–12) and the mean was 7.96 (standard error 0.21). Six out of 109 flocks (5.5%) had a value of 4 (the minimum), whereas 7 (6.4%) flocks had a value of 12 (the maximum). Nineteen out of 30 (63.3%) at the top score (≥10) were “pastoralists only” with a higher parturition rate (median = 0.70, range 0.40–2.73 for sheep; and median = 0.80, range 0.19–1.50 for goats), and higher multiplication rate (median = 1.14, range 0.58–1.43 for sheep; and median = 1.18, range 0.73–1.57 for goats) than those 34 pastoralists with the lowest FEI values (score ≤ 6).

Those pastoralists with the lowest FEI were a mixture of “pastoralists only” (15/34, 44.1%) and “pastoralists and traders”, characterized by a very low parturition rate (median = 0.39, range 0.08–1.00 in sheep and median = 0.34, range 0.07–0.73 in goats) and low multiplication rate (median = 0.88, range 0.38–1.17 in sheep and median = 0.90, range 0.21–1.24 in goats). Those flocks with the lowest FEI had significantly lower parturition rate (Kruskal–Wallis rank sum test, *p*-value <0.001in sheep and goats) and lower multiplication rate (Kruskal–Wallis rank sum test, *p*-value <0.001 in sheep and goats) compared with those with the highest score

There was no evidence of a difference in number of small ruminants, number of sheep and goats or other parameters (excepting those parameters used to create the score) found between pastoralists with high and low FEI scores.

The FEI was tested for Normality by Shapiro–Wilk test and the null hypothesis was rejected (*p*-value = 0.001). The FEI was positively correlated with the multiplication rate in sheep (*r* = 0.35; *p*-value <0.001) and goats (*r* = 0.4; *p*-value <0.001). No correlation was found between the FEI and the net production rate using Spearman's rho test.

The FEI was not normally distributed in either “pastoralists and traders” (Shapiro–Wilk test *p*-value = 0.04) or “pastoralists only” (Shapiro–Wilk test *p*-value = 0.02); therefore, a Kruskal–Wallis rank sum test was used for a more detailed exploration. The “pastoralists and traders” group had significantly lower FEI score (median = 7.5, IQR: 6–9) compared with “pastoralists only” flocks (median = 8, IQR 6–10) (Kruskal–Wallis rank sum test, *p*-value <0.001; Fig. 4).

## 4 Discussion

The main objective of this research was to test a novel approach for capturing data and using those data to calculate different production and reproduction parameters in pastoralist communities. Our questionnaire worked for 92% of the pastoralists interviewed. For the few pastoralists for whom data could not be captured, this was caused by difficulties of reporting the actual number of animals in each age and sex category. These results indicate that the questionnaire is suitable for use in future programmes and interventions.

Studies done using a similar methodology in Kenya, or other East African country, were not found, therefore our results were compared with studies that used a similar methodology (retrospective survey) in other settings such as Senegal (Ejlertsen, Poole & Marshall, 2012a), Mali (Diawara, Hiernaux, Mougin, Gangneron & Soumaguel, 2017; Ejlertsen, Poole & Marshall, 2012b) and The Gambia (Marshall et al., 2011). Additionally, our results were compared with other methodologies using the systematic review conducted by Otte and Chilonda (2002). Those studies reported the mean and standard deviation of the parameters; thus, although our parameters were not normally distributed, those summary statistics have been included in Tables 3 and 4, to allow comparison. In general, the figures obtained in our study were lower for reproductive parameters, particularly parturition, fecundity and net fecundity rate, and similar for the rest of the parameters, although a high variability was also found in the literature. Parturition rate in this study was slightly higher in sheep compared with goats. Although this is not a common finding, it has been reported in previous studies (Ndamukong, 1987).

The differences between values reported in this study compared with previously published data could in part be explained by the fact that different sheep and goat populations are being evaluated. Higher variability may also be explained because of the simplicity of our study, which calculates overall indicators. We calculate reproductive indicators using one age-category of reproductive females as a denominator, as opposed to other methodologies which stratify the population in yearly age groups. In summary, comparing parameters from different studies or based on literature data should be interpreted with caution, as other authors have previously pointed out (Lesnoff, 2015).

The parameters calculated were not normally distributed; the distributions of most of them were right skewed with the presence of some outliers (data distribution is shown in Fig. 2 and annex 3 of the supplementary material). We decided to incorporate those outliers since we consider that deleting them or replacing them with a value less extreme could bias the estimates and, as our approach is based on the farmer's recall, we consider that all answers obtained have the same trustworthiness. Furthermore, robust summary statistics were reported so their influence is almost negligible.

A direct question to pastoralists about whether they usually buy animals to bring into their flock(s) was asked. This question was used as a proxy to understand whether some pastoralists were usually involved in trading activities and therefore classify them as “pastoralists only” or “pastoralists and traders”. *Post-hoc* analysis showed that 91.2% (62/68) of “pastoralists only” did not purchase any animal (or only one) in the previous 12 months (60 did not purchase any animal during the study period), whereas 93.5% (58/62) “pastoralists and traders” bought animals during the previous 12 months (56 of them bought more than one animal during the study period), validating the use of this direct question.

Overall, compared with other populations, the pastoralist system in Kajiado was characterized by low reproductive rates, low offtake rates and very low intake rates. Those findings were consistent with other reports (Marshall et al., 2011) and they should be interpreted considering the long-time survival strategy of Maasai pastoralists. Offtake and intake rates need to be understood in connection with accumulation, as animals are accumulated in good years as a survival strategy (Bekure et al., 1991). In the present study, 48 pastoralists had increased the number of sheep and goats in their flocks. It should be noted than 81.3% of them were “pastoralists only”. While 37 pastoralists decreased the number of sheep and goats in their flocks, 75.7% of them were “pastoralists and traders”. This shows the different strategy followed by those two types of pastoralists, with “pastoralists only” increasing the size of their flocks during the 12 month study period.

The exploration of the relationship between variables revealed some interesting findings: for example, dystocia rate was positively correlated with net production rate. A potential explanation is that larger body size is related to a higher market price. Pastoralists with a higher net production rate and more market-oriented mindset, may be selecting animals with larger body size. When big males are used as breeding (e.g. Dorper males with Red Maasai females or crosses) this can cause problems. Also, dystocia and abortion rate were correlated with mortality rate, which may indicate that diseases are present, reflecting bad performance.

A Flock Efficiency Indicator is proposed to measure efficiency of pastoralist small ruminant flocks, allowing detection of those that exhibit poor performance. This indicator was positively correlated with multiplication rate, implying that better performance supported pastoralists to sustain and increase herd size. Yet, this performance indicator was not correlated with net production rate (that measures the balance between offtakes and intakes). This implies that pastoralists exhibiting poor performance in their flocks will aim to compensate by purchasing higher number of animals, while good performers may not prioritize offtake, in order to increase flock size and therefore its value. Furthermore, the relationship between the FEI and the type of pastoralist (“pastoralists only” or “pastoralists and traders”) provides an interesting insight into the strategy followed by these two groups. “Pastoralists only” are more oriented to good flock performance with higher productive indicators and a tendency to increase flock size, mainly because the number of births is higher than the number of deaths. This also suggests that “pastoralists and traders” may be more focused on buying and selling animals, than on managing flock performance.

A retrospective approach based on farmer recall of a flocks’ demography, has been extensively used (the earliest written evidence found is from 1975). This methodology was regularly revisited and improved through different projects implemented in West Africa (Lesnoff & Lancelot, 2009; Lesnoff, 2011, Lesnoff et al., 2016). Our approach used this methodology as a preliminary step. However, during the fieldwork it was not feasible to capture data about flock exits and entries at 12 month age intervals (i.e., age class 0, ages “0 to 12 months”, age class 1, ages “>12 to 24 months” and so on). Hence, we initially developed and tested an approach using three age group categories of animals, which did not work and was not accepted by pastoralists. Thus, we decided to use an approach with two age categories, using 2 years as the cut-off age to calculate number of reproductive animals in the flock because pastoralists change their animals’ management when they are older than 2 years old. *Post-hoc* analysis of age at first parity showed that the mean age for ewes was 2.06 and for does was 2.23, validating the assumption used in our methods. However, it is common for flocks to have some reproductive females less than 2 years old. Since this cut-off was used for all flocks, it is assumed that any bias was systematic across the parameters measured, with the exception of any flocks with a very different age structure (i.e., results are not comparable for flocks where there is a large number of reproductive females ≤2 years old compared with >2 years old). Our results should be interpreted with caution when comparing with those from other studies that use an exact number of reproductive females to calculate reproductive parameters. Furthermore, pastoralists provided the number of animals per category present in the flock at the time of the survey; the enumerators did not perform any inspection of the animals or animals’ teeth to provide better estimates. Thus, our approach is entirely based on the pastoralists’ recall.

Our questionnaire was able to obtain overall estimates and reproductive indicators as well as an FEI. The main advantage of our approach is its suitability for any type of flock, including nomadic ones. Thus, it is particularly suitable for fieldwork and use in large benchmarking, research or surveillance programmes, and with a large variety of stakeholders.

Data captured would have been impossible to obtain under the same conditions by other means within the time frame available. Other methodologies are more time consuming and require more engagement with the pastoralists and their flocks (including regular visits of the enumerators). This can potentially cause participants to refuse to participate or finish the questionnaire, as observed during the piloting and development of this study. In this regard, it should be noted that our methodology was well accepted by pastoralists Thus, it can be recommended as a rapid assessment tool, providing a basis for follow-up of flocks over time and assessing year-by-year variation.

However, since the tool's accuracy may be lower than other methods, further studies could be performed. For example implementing different approaches for the same flock (such as long-term monitoring methods) would allow further validation of the tool and better quantify the errors; or using a similar questionnaire in the following year would enable calculation of the flock size 12 months ago and incorporation of those recall errors and ensuing uncertainty into performance indicator calculations.

Several other limitations are present in this study. The data collected are based on pastoralist recall for the 12 month period before the survey and on their knowledge of the number of animals present at the time of the survey. Thus, it is likely to contain a certain level of error due to recall bias. Outlier values were included in the analysis since their trustworthiness should be considered comparable to the rest of the values reported by interviewees. These results relate to the specific environmental conditions of the 12 month period prior to data collection; annual differences can have a significance impact on flock parameters such as reproductive performance (Blackburn & Field, 1990; Marshall et al., 2011). Variation of values between years was not captured.

This methodology can be applied in flocks of a specific size, with the recall being easier for flocks with smaller flock sizes. It is however important to note that the methodology was suitable for all the pastoralists flocks in Kajiado. In the present study, 16 flocks had more than 200 small ruminants and 28 of them had between 200 and 100 animals. There was no that a larger flock size was associated with poor questionnaire recall performance.

Although participants were not randomly selected, they were believed to be representative of the population. Husbandry management practices in pastoralist flocks from the same area tend to be similar and animals often graze together, thus adverse events and diseases will affect them in a similar way. Therefore, the values of the parameters obtained are not completely independent.

## 5 Conclusion

Our methodology enables the capture of small ruminant flock performance data from pastoralists that allows identification of pastoralists with lower productivity, who are therefore more vulnerable. In addition, we propose an overall flock level indicator, the FEI that can be used to assess the combined efficiency of both species for these pastoralists. The methodology used was easy to implement and acceptable to pastoralists, with very low levels of rejection.

The tool proposed in this study can be used to obtain benchmarking data from pastoralists over time. It can be used by stakeholders and policy makers to assess the effectiveness of their interventions. Moreover, the results generated represent a baseline indicator for future research and economic studies needed to assess the viability of pastoralist systems. Future studies will be conducted to determine the risk factors and management characteristics of pastoralists with poor and good performance, and assess the economic benefits or losses associated with different performance levels.

## Availability of data and materials

Data used in the study can be made available upon request to the corresponding author.

## Consent for publication

Not applicable.

## Ethical approval

The study received ethical approval from the International Livestock Research Institute under the reference ILRI-IREC2019–18 and from the Social Sciences Research Ethical Review Board from Royal Veterinary College, reference URN SR2019–0236.

## Author details

Veterinary Epidemiology, Economics and Public Health, Department of Pathobiology and Population Sciences, The Royal Veterinary College, Hawkshead Lane, North Mymms, Hatfield, Herts, AL9 7TA, UK.

## Ethical statement

Hereby, I, Cristina Ballesteros, assure that for the manuscript *“Development of a tool for rapid assessment of reproduction and production efficiencies of small ruminants pastoralist flocks and its application in Kajiado, Kenya”* the following is fulfilled: All procedures in this study received ethical approval from the International Livestock Research Institute under the reference ILRI-IREC2019–18 and from the Social Sciences Research Ethical Review Board from Royal Veterinary College, reference URN SR2019–0236.

## Declaration of Competing Interest

The authors declare that they have no known competing financial interests or personal relationships that could have appeared to influence the work reported in this paper.

## Appendix Supplementary materials

Image, application 1
