# Supplementary material for: Developing and validating a rapid assessment tool for small ruminant reproduction and production in pastoralist flocks in Kajiado, Kenya
Source: Vet Anim Sci. 2021 Jun 17;13:100186. doi: 10.1016/j.vas.2021.100186 (PMC8242044; doi:10.1016/j.vas.2021.100186)

**Supplementary Information for:**

**“Development of a tool for rapid assessment of reproduction and production efficiencies of small ruminants pastoralist flocks and its application in Kajiado, Kenya”**

**Annex 1: Questionnaire**

1. **Flock identification number** (FlockID) Unique identification number for each flock
2. **Interviewer** (Single-answer question)
3. **Date of interview (DD/MM/YYY)** (automatically recorded with the tablet)
4. **GPS codes** (automatically recorded with the tablet)

| Latitude |  |
| --- | --- |
| Longitude |  |

1. **Sub-county** (Single-answer question)

| Kajiado East |  |
| --- | --- |
| Kajiado Central |  |
| Kajiado West |  |

1. **Ward** (Single-answer question)

| Ildamat (Kajiado Central) |  |
| --- | --- |
| Matapato South (Kajiado Central) |  |
| Kaputiei North (Kajiado East) |  |
| Kenyawa-Poka (Kajiado East) |  |
| Magadi (Kajiado West) |  |

1. **Household head contact details.**

**Respondent's name**

|  |
| --- |

**Phone number**

|  |
| --- |

1. **Do you usually buy animals to bring into your flock(s)?**

| Yes |  |
| --- | --- |
| No |  |

1. **What is the main reason for keeping shoats?** * (**Select as appropriate and rank**)

| regular cash income |  |
| --- | --- |
| insurance against emergencies |  |
| milk production |  |
| celebrations |  |
| prestige |  |
| drought tolerance |  |
| disease resistance |  |
| other reasons, please specify |  |
| no more answers provided |  |

1. **Are we in a manyatta?**

| Yes |  |
| --- | --- |
| No |  |

1. *If the answer is “yes” in Q10, the following information will be captured:* **Total of sheep and goats in the manyatta**

|  | shoats |
| --- | --- |

1. *If the answer is “yes” in Q10, the following information will be captured:* **Of these, how many are sheep?**

|  | sheep |
| --- | --- |

1. *If the answer is “yes” in Q10, the following information will be captured:* **Of those, how many are goats?**

|  | goats |
| --- | --- |

1. **How many flocks of sheep and goats do you have? (flock: group of sheep and goats raised in different locations)**

| Total flocks |  |
| --- | --- |

1. **How many SHEEP do you have now in all your flocks?**

| Total sheep |  |
| --- | --- |

1. **How many GOATS do you have now in all your flocks**

| Total Goats |  |
| --- | --- |

1. We are now going to distribute those sheep, goats and cattle in different compartments depending on their age and sex. **How many of those sheep fall in each of the following categories?**

| Young females ≤ 2 years |  |
| --- | --- |
| Young males ≤ 2 years |  |
| Ewes > 2 years old |  |
| Rams > 2 years old |  |
| Breeding rams > 2 years old |  |

1. **How many of those goats fall in each of the following categories?**

| Young females ≤ 2 years |  |
| --- | --- |
| Young males ≤ 2 years |  |
| Does > 2 years old |  |
| Bucks > 2 years old |  |
| Breeding bucks > 2 years old |  |

1. **What are the breeds of sheep present in the flock(s)?** RANK question

| Red Maasai |  |
| --- | --- |
| Dorper |  |
| Somali |  |
| Other breed(s), please specify |  |
| Unknown |  |
| I don’t have (sheep) |  |

1. **What are the breeds of goats present in the flock(s)?** RANK question

| Galla |  |
| --- | --- |
| Small East Africa |  |
| Alpine |  |
| Other breed(s), please specify |  |
| Unknown |  |
| I don’t have (goats) |  |

1. **To the best of your knowledge, how many lambs were born alive during the last 12 months?**

|  | lambs |
| --- | --- |

1. **Are there other LAMBS that were born ALIVE DURING THE LAST 12 MONTHS BUT DIED NOT INCLUDED IN THE NUMBER ABOVE??**

|  | lambs |
| --- | --- |

1. **To the best of your knowledge, how many kids were born alive during the last 12 months?**

|  | kids |
| --- | --- |

1. **Are there other KIDS that were born ALIVE DURING THE LAST 12 MONTHS BUT DIED NOT INCLUDED IN THE NUMBER ABOVE??**

|  | kids |
| --- | --- |

1. **To the best of your knowledge, how many LAMBS were BORN DEAD during the last 12 months?**

|  | Lambs |
| --- | --- |

1. **To the best of your knowledge, how many KIDS were BORN DEAD during the last 12 months?**

|  | Kids |
| --- | --- |

1. **How many ewes gave TWIN BIRTHS during the last 12 months?**

|  | Twin births sheep |
| --- | --- |

1. **Of these births, how many LAMBS were born DEAD?**

|  | Lambs born dead |
| --- | --- |

*Note: This will allow estimating correctly the number of females given births in the last 12 months (takes into account that born dead could come from single parity or twin parities)

1. **How many does gave TWIN BIRTHS during the last 12 months?**

|  | Twin births does |
| --- | --- |

1. **Of these births, how many KIDS were born DEAD?**

|  | Kids born dead |
| --- | --- |

*Note: This will allow estimating correctly the number of females given births in the last 12 months (takes into account that born dead could come from single parity or twin parities)

1. **How many does gave TRIPLET BIRTHS during the last 12 months?**

|  | Triplet births does |
| --- | --- |

1. **Of these births, how many KIDS were born DEAD?**

|  | Kids born dead |
| --- | --- |

*Note: This will allow estimating correctly the number of females given births in the last 12 months (takes into account that born dead could come from single parity, twin or triplet parities)

1. **How many ABORTIONS did you notice during the last 12 months in your EWES?**

|  | abortions |
| --- | --- |

1. **How many ABORTIONS did you notice during the last 12 months in your DOES?**

|  | abortions |
| --- | --- |

1. **How many DIFFICULT BIRTHS did you notice during the last 12 months in your EWES?**

|  | Difficult births |
| --- | --- |

1. **How many DIFFICULT BIRTHS did you notice during the last 12 months in your DOES?**

|  | Difficult births |
| --- | --- |

1. **What is the normal age at which your EWES have the FIRST LAMB?** (Tick as appropriate)

| ≤1.5 years |  |
| --- | --- |
| ≤ 2 years |  |
| ≤ 2.5 years |  |
| ≤ 3 years |  |
| ≤ 3.5 years |  |
| ≤ 4 years |  |
| I don’t have (sheep) |  |

1. **What is the normal age at which your DOES have the FIRST KID?** (Tick as appropriate)

| ≤1.5 years |  |
| --- | --- |
| ≤ 2 years |  |
| ≤ 2.5 years |  |
| ≤ 3 years |  |
| ≤ 3.5 years |  |
| ≤ 4 years |  |
| I don’t have (goats) |  |

1. **Do you remember at what age do you usually REMOVE from the flock your OLDEST EWE?**

|  | Age (years) |
| --- | --- |
| Ewe |  |

1. **How many times does a normal EWE give birth in her lifetime in your flock(s)**

|  |
| --- |

1. **Do you remember at what age do you usually remove from the flock your OLDEST DOE?**

|  | Age (years) |
| --- | --- |
| Doe |  |

1. **How many times does a normal doe give birth in her lifetime in your flock(s)**

|  |
| --- |

1. **What characteristic do you consider most important when selecting and keeping your sheep and goats?** (free text)

|  |
| --- |

**EXITS:**

1. **How many sheep and goats left the flock(s) during the last twelve months? SALE or BARTER**

|  | Number |
| --- | --- |
| Total sheep SOLD or BARTERED |  |
| Total goats SOLD or BARTERED |  |

1. **Sale age categorization in sheep**

|  | Number |
| --- | --- |
| Sale: Young females (sheep) ≤ 2 years |  |
| Sale: Young males (sheep) ≤ 2 years |  |
| Sale: Ewes older than 2 years |  |
| Sale: Rams older than 2 years |  |

1. **Sale age categorization in goats**

|  | Number |
| --- | --- |
| Sale: Young females (goats) ≤ 2 years |  |
| Sale: Young males (goats) ≤ 2 years |  |
| Sale: Does older than 2 years |  |
| Sale: Bucks older than 2 years |  |

1. **What were the main reason(s) to sell/barter those sheep and goats?** (rank question)

| To purchase food for household |
| --- |
| To pay school fees |
| To purchase other household needs |
| To improve your flock or have capital to invest in your flock |
| Other, please specify |
| No more answers were provided |

1. **How many sheep and goats left the flock(s) during the last twelve months? SLAUGHTER FOR OWN CONSUMPTION**

|  | Number |
| --- | --- |
| Total sheep SLAUGHTERED FOR OWN CONSUMPTION |  |
| Total goats SLAUGHTERED FOR OWN CONSUMPTION |  |

1. **Slaughter age categorization in sheep**

|  | Number |
| --- | --- |
| Slaughter: Young females (sheep) ≤ 2 years |  |
| Slaughter: Young males (sheep) ≤ 2 years |  |
| Slaughter: Ewes older than 2 years |  |
| Slaughter: Rams older than 2 years |  |

1. **Slaughter age categorization in goats**

|  | Number |
| --- | --- |
| Slaughter: Young females (goats) ≤ 2 years |  |
| Slaughter: Young males (goats) ≤ 2 years |  |
| Slaughter: Does older than 2 years |  |
| Slaughter: Bucks older than 2 years |  |

1. **How many sheep and goats left the flock(s) during the last twelve months? GIFT/DOWRY/INHERITANCE**

|  | Number |
| --- | --- |
| Total sheep exited the flock as a GIFT/DOWRY/INHERITANCE |  |
| Total goats exited the flock as a GIFT/DOWRY/INHERITANCE |  |

1. **Gift age categorization in sheep**

|  | Number |
| --- | --- |
| Gift: Young females (sheep) ≤ 2 years |  |
| Gift: Young males (sheep) ≤ 2 years |  |
| Gift: Ewes older than 2 years |  |
| Gift: Rams older than 2 years |  |

1. **Gift age categorization in goats**

|  | Number |
| --- | --- |
| Gift: Young females (goats) ≤ 2 years |  |
| Gift: Young males (goats) ≤ 2 years |  |
| Gift: Does older than 2 years |  |
| Gift: Bucks older than 2 years |  |

1. **How many sheep and goats left the flock(s) during the last twelve months?LOST**

|  | Number |
| --- | --- |
| Total sheep exited the flock LOST |  |
| Total goats exited the flock LOST |  |

1. **Lost age categorization in sheep**

|  | Number |
| --- | --- |
| Lost: Young females (sheep) ≤ 2 years |  |
| Lost: Young males (sheep) ≤ 2 years |  |
| Lost: Ewes older than 2 years |  |
| Lost: Rams older than 2 years |  |

1. **Lost age categorization in goats**

|  | Number |
| --- | --- |
| Lost: Young females (goats) ≤ 2 years |  |
| Lost: Young males (goats) ≤ 2 years |  |
| Lost: Does older than 2 years |  |
| Lost: Bucks older than 2 years |  |

1. **How many sheep and goats left the flock(s) during the last twelve months? FOR ANY OTHER REASONS**

|  | Number |
| --- | --- |
| Total sheep exited the flock for any OTHER REASON |  |
| Total goats exited the flock for any OTHER REASON |  |

1. **“Other reason” age categorization in sheep**

|  | Number |
| --- | --- |
| Other: Young females (sheep) ≤ 2 years |  |
| Other: Young males (sheep) ≤ 2 years |  |
| Other: Ewes older than 2 years |  |
| Other: Rams older than 2 years |  |

1. **“Other reason” age categorization in goats**

|  | Number |
| --- | --- |
| Other: Young females (goats) ≤ 2 years |  |
| Other: Young males (goats) ≤ 2 years |  |
| Other: Does older than 2 years |  |
| Other: Bucks older than 2 years |  |

1. **DEAD/CULLED How many animals died due to DISEASES during the last 12 months? Natural dead or culled, excluding slaughtering for own consumption.**

|  | Number |
| --- | --- |
| Total SHEEP dead due to DISEASES |  |
| Total GOATS dead due to DISEASES |  |

1. **Disease: age distribution in sheep**

|  | Number |
| --- | --- |
| Disease: Young females (sheep) ≤ 2 years |  |
| Disease: Young males (sheep) ≤ 2 years |  |
| Disease: Ewes older than 2 years |  |
| Disease: Rams older than 2 years |  |

1. **Disease: age categorization in goats**

|  | Number |
| --- | --- |
| Disease: Young females (goats) ≤ 2 years |  |
| Disease: Young males (goats) ≤ 2 years |  |
| Disease: Does older than 2 years |  |
| Disease: Bucks older than 2 years |  |

1. **How many SHEEP and GOATS died during the last twelve months because they were PREDATED?**

|  | Number |
| --- | --- |
| Total SHEEP dead due to PREDATION |  |
| Total GOATS dead due to PREDATION |  |

1. **Predation: age distribution in sheep**

|  | Number |
| --- | --- |
| Predation: Young females (sheep) ≤ 2 years |  |
| Predation: Young males (sheep) ≤ 2 years |  |
| Predation: Ewes older than 2 years |  |
| Predation: Rams older than 2 years |  |

1. **Predation: age categorization in goats**

|  | Number |
| --- | --- |
| Predation: Young females (goats) ≤ 2 years |  |
| Predation: Young males (goats) ≤ 2 years |  |
| Predation: Does older than 2 years |  |
| Predation: Bucks older than 2 years |  |

1. **How many SHEEP and GOATS died during the last twelve months because of a DROUGHT?**

|  | Number |
| --- | --- |
| Total SHEEP dead as a result of a DROUGHT |  |
| Total GOATS dead as a result of a DROUGHT |  |

1. **Drought: age distribution in sheep**

|  | Number |
| --- | --- |
| Drought: Young females (sheep) ≤ 2 years |  |
| Drought: Young males (sheep) ≤ 2 years |  |
| Drought: Ewes older than 2 years |  |
| Drought: Rams older than 2 years |  |

1. **Drought: age categorization in goats**

|  | Number |
| --- | --- |
| Drought: Young females (goats) ≤ 2 years |  |
| Drought: Young males (goats) ≤ 2 years |  |
| Drought: Does older than 2 years |  |
| Drought: Bucks older than 2 years |  |

1. **How many SHEEP and GOATS died during the last twelve months for any OTHER reason?**

|  | Number |
| --- | --- |
| Total sheep dead for any OTHER reason |  |
| Total goats dead for any OTHER reason |  |

1. **“Other reason”: age distribution in sheep**

|  | Number |
| --- | --- |
| Other: Young females (sheep) ≤ 2 years |  |
| Other: Young males (sheep) ≤ 2 years |  |
| Other: Ewes older than 2 years |  |
| Other: Rams older than 2 years |  |

1. **“Other reason” age categorization in goats**

|  | Number |
| --- | --- |
| Other: Young females (goats) ≤ 2 years |  |
| Other: Young males (goats) ≤ 2 years |  |
| Other: Does older than 2 years |  |
| Other: Bucks older than 2 years |  |

1. **What adverse events affected your flock(s) during the last 12 months?** (multiple response question)

| None |  |
| --- | --- |
| Drought |  |
| Heat stress |  |
| Flooding |  |
| Disease |  |
| Other, please specify |  |

**ENTRIES:**

1. **How many SHEEP and GOATS entered the flock(s) during the last twelve months? PURCHASE or BARTER**

|  | Number |
| --- | --- |
| Total sheep PURCHASED or BARTERED |  |
| Total GOATS PURCHASED or BARTERED |  |

1. **Purchased age categorization in sheep**

|  | Number |
| --- | --- |
| Purchase: Young females (sheep) ≤ 2 years |  |
| Purchase: Young males (sheep) ≤ 2 years |  |
| Purchase: Ewes older than 2 years |  |
| Purchase: Rams older than 2 years |  |

1. **Purchased age categorization in goats**

|  | Number |
| --- | --- |
| Purchase: Young females (goats) ≤ 2 years |  |
| Purchase: Young males (goats) ≤ 2 years |  |
| Purchase: Does older than 2 years |  |
| Purchase: Bucks older than 2 years |  |

1. **How many sheep and goats entered the flock(s) during the last twelve months? GIFT/DOWRY/INHERITANCE**

|  | Number |
| --- | --- |
| Total sheep entered the flock as a GIFT/DOWRY/INHERITANCE |  |
| Total goats entered the flock as a GIFT/DOWRY/INHERITANCE |  |

1. **Gift age categorization in sheep**

|  | Number |
| --- | --- |
| Gift: Young females (sheep) ≤ 2 years |  |
| Gift: Young males (sheep) ≤ 2 years |  |
| Gift: Ewes older than 2 years |  |
| Gift: Rams older than 2 years |  |

1. **Gift age categorization in goats**

|  | Number |
| --- | --- |
| Gift: Young females (goats) ≤ 2 years |  |
| Gift: Young males (goats) ≤ 2 years |  |
| Gift: Does older than 2 years |  |
| Gift: Bucks older than 2 years |  |

1. **How many sheep and goats entered the flock(s) during the last twelve months? OTHER REASONS**

|  | Number |
| --- | --- |
| Total sheep entered the flock for any OTHER REASON |  |
| Total goats entered the flock for any OTHER REASON |  |

1. **“Other reason” age categorization in sheep**

|  | Number |
| --- | --- |
| Other: Young females (sheep) ≤ 2 years |  |
| Other: Young males (sheep) ≤ 2 years |  |
| Other: Ewes older than 2 years |  |
| Other: Rams older than 2 years |  |

1. **“Other reason” age categorization in goats**

|  | Number |
| --- | --- |
| Other: Young females (goats) ≤ 2 years |  |
| Other: Young males (goats) ≤ 2 years |  |
| Other: Does older than 2 years |  |
| Other: Bucks older than 2 years |  |

1. **What are your main sources of income?** (Rank question)

| Small-ruminant rearing |
| --- |
| Cattle rearing |
| Crop farming |
| Trading livestock products |
| Trading crop products |
| Other business (neither related with livestock nor with crops) |
| Other |
| No more answers were provided |

**Annex 2: Formulae used to calculate the production parameters:**

**Parturition rate** ($r_{partutition}$)

$$r_{partutition}=\frac{n_{parturition}}{N_{reproductive females}}$$

$n_{parturition}$ corresponds to the numbers of parturitions recorded at the date of the survey. It was obtained through the number of animals born alive, animals born dead, and considering twin births reported and triplet births (in goats).

The number of reproductive females ($N_{reproductive females}$) was the number of reproductive females present at the moment of the survey (Lesnoff, 2009)

**Prolificacy rate** ($prol$)

$$prol =\frac{b}{n_{parturitions}}$$

Where $b$ is the number of offspring (stillborn and alive) obtained during the year and $n_{parturitions}$ is the number of parturitions recorded during the study period. It includes stillbirths.

**Twinning rate** ($r_{twinning}$*)*

$$r_{twinning}= \frac{n_{twin}}{n_{parturitions}}$$

Where $n_{twin}$ is the number of twin parturtions during the study period and $n_{parturitions}$is the total number of parturitions during the study period.

**Triplet rate** *(*$r_{triplet}$*, only goats)*

$$r_{triplet}= \frac{n_{triplet}}{n_{parturitions}}$$

Where $n_{triplet}$is the number of triplet parturitions during the study period and $n_{parturitions}$ is the total number of parturitions during the study period.

***Fecundity rate*** ($r_{fecundity}$)

$$r_{fecundity}=\frac{b}{N_{reproductive females}}$$

Where $b$ is the number of offspring (stillborn and alive) obtained during the year and $N_{reproductive females}$is the number of reproductive females present during the year.

**Abortion rate** ($r_{abortion}$)

$$r_{abortion}=\frac{n_{abortion}}{N_{reproductive females}}$$

Where $n_{abortion}$ corresponds to the numbers of abortions recorded at the date of the survey.

**Stillbirth rate** ($r_{stillbirth}$)

$$r_{stillbirth}=\frac{n_{stillbirth}}{b}$$

Where $n_{stillbirth}$ is the number of stillborns and $b$ is the number of offsprings (stillborn and alive) obtained during the 12-month period.

**Dystocia rate** ($r_{dystocia}$)

$$r_{dystocia}=\frac{n_{dystocia}}{n_{parturitions}}$$

Where $n_{dystocia}$ corresponds to the numbers of obstructed labour recorded at the date of the survey during the year and $n_{parturitions}$ is the number of parturitions during that year*.*

**Net prolificacy rate** ($r_{NET\_PROL}$)

$$r_{NET\_PROL}=\frac{b_{alive}}{n_{parturition}}$$

Where $b_{alive}$ is the number of offspring born alive obtained during the 12-month period and $n_{parturitions}$ is the number of parturitions during that year.

**Net fecundity rate** ($r_{NET\_FEC}$)

$$r_{NET\_FEC}=\frac{b_{alive}}{N_{reproductive females}}$$

Where $b_{alive}$ is the number of offspring born alive obtained during the 12-month period and $N_{reproductive females}$ is the number of reproductive females present during the year.

**Multiplication rate** ($R_{m}$)

$$R_{m}=\frac{herd size at date of survey}{herd size 12 months before}$$

A value of >1 indicates a positive growth rate in the year.

**Growth rate** ($R_{g}$)

$$R_{g}=100*(R_{m}-1)$$

**Production rate** ($R_{p}$)

$$R_{p}=\frac{(\Delta_{n}+O-I)}{N}$$

Where $\Delta_{n}$ represents the flock variation during the last twelve months, $O$ is the number of offtake over the year and $I$ is the number of intake over the year. $N$is the mean flock size expressed as number of animal-year at risk during the study period. An approximate calculation of the mean flock size was obtained subtracting entries and adding exits to the final number of animals in the flock to obtain the initial number of animals at the beginning of the 12-month period, and obtaining the arithmetic mean between flock sizes at the beginning and the end of the study-period (FAO, 2018; Lessnoff, 2015). It was obtained by approximate calculation (Dohoo et al., 2009).

$\Delta_{n}, O \mathrm{and}I$ can be expressed in different units, however if they are expressed in number of animals, $R_{p}$is the numerical performance.

As:

$$\Delta_{n}=B-D-(O-I)$$

$R_{p}$calculation can be simplified as:

$$R_{p}=\frac{(B-D)}{N}$$

**Exploitation rate or net production rate** ($R_{net}$)

$$R_{net}=\frac{(O-I)}{N}$$

**Mortality rate** ($r_{mortality}$)

$$r_{mortality}=\frac{d}{N}$$

$d$ is the number of natural deaths that occurred in the category during the study period and $N$is mean flock size during the study period.

**Intake rate** ($r_{intake}$)

$$r_{intake}=\frac{d_{intake}}{N}$$

$d_{intake}$ is the number of is the number of animals that entered the herd during the study period and $N$is mean flock size during the study period.

**Offtake rate** ($r_{offtake}$)

$$r_{offtake}=\frac{d_{offtake}}{N}$$

$d_{offtake}$ is the number of animals that left the herd as offtake during the study period $N$is the mean flock size during the study period.

**Annex 3:** **Histogram and boxplot of the productive parameters**


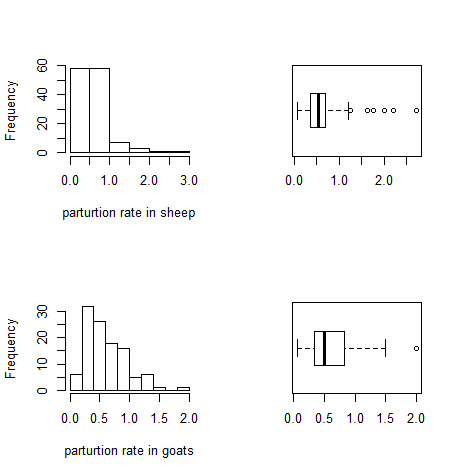

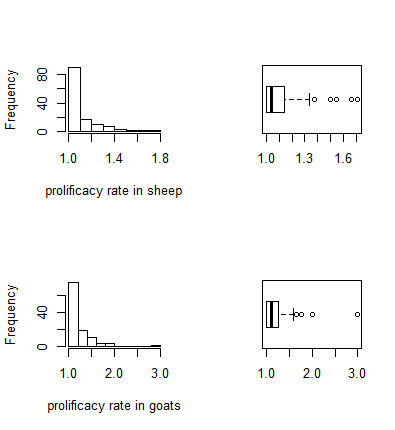


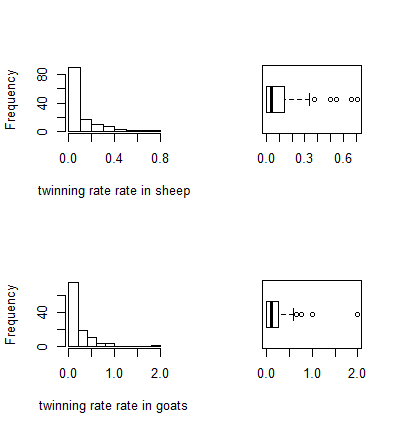

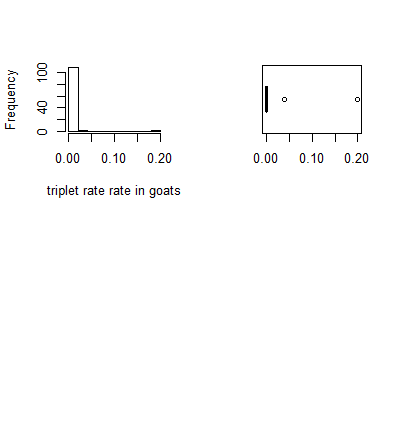


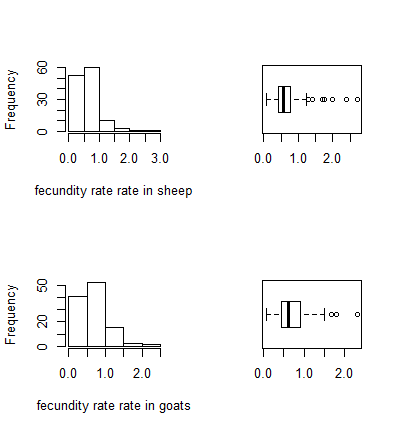

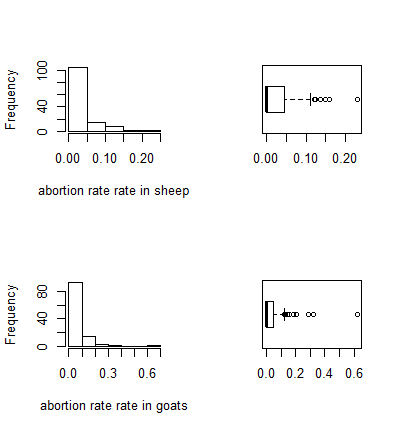


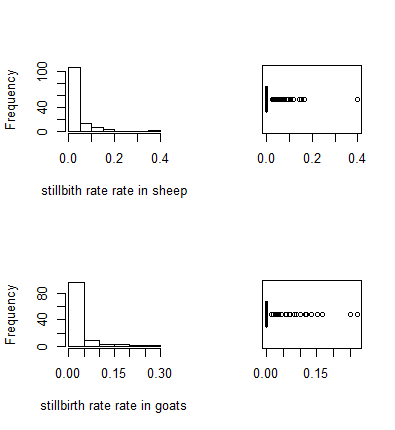

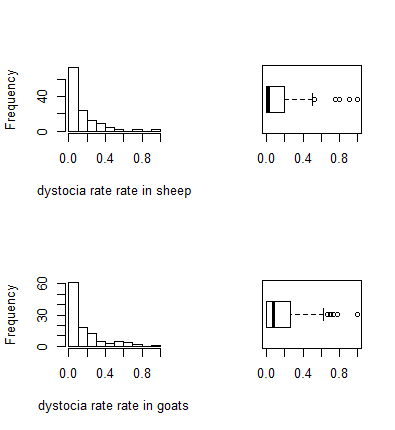


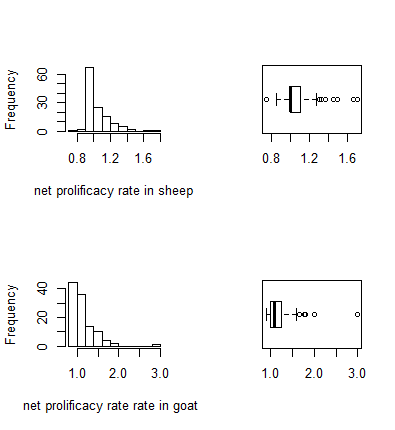

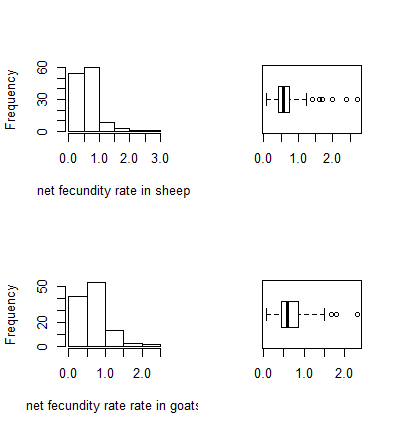


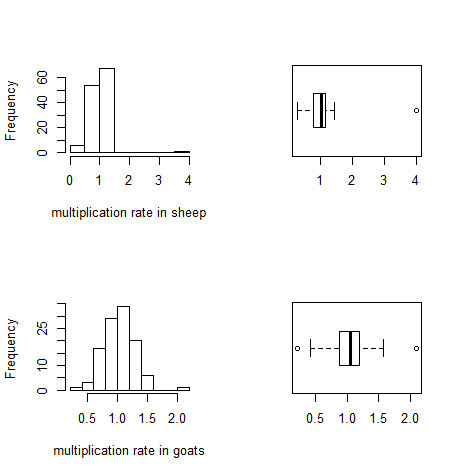

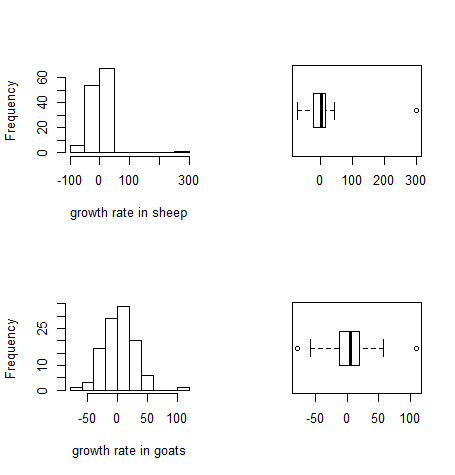


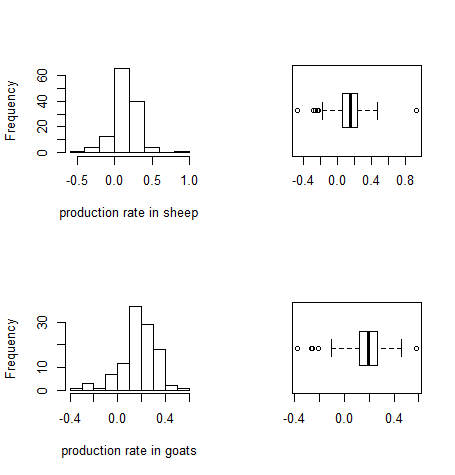

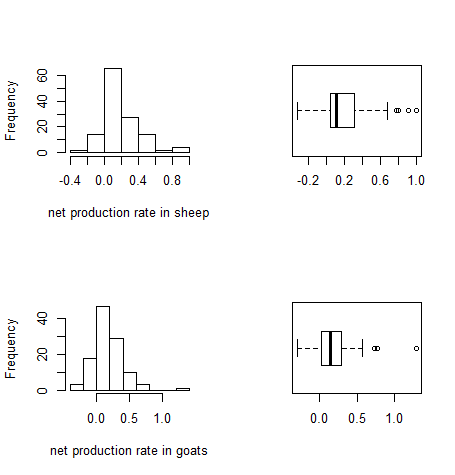


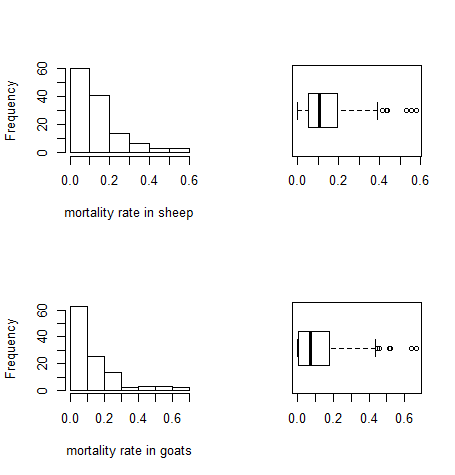

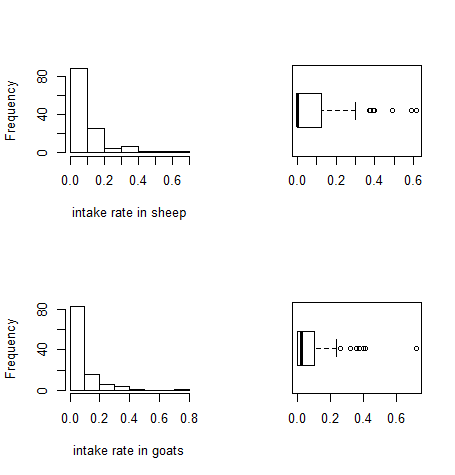


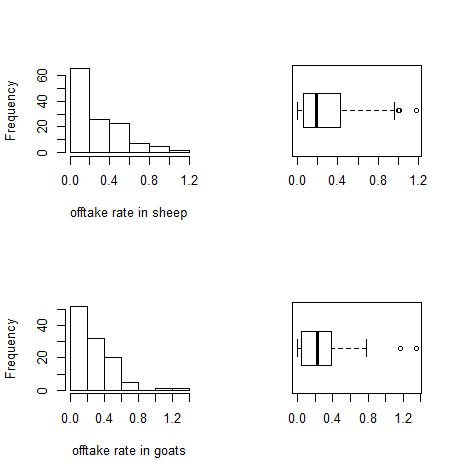


**Annex 4: Principal component analysis (PCA) results**

A PCA was performed to identify patterns and reduce the dimensionality of the data but minimising information loss. Due to the positively skewness of the variables a logarithmic transformation was done before PCA analysis computing log(1+x).

Components with an eigenvalue >1, showing that principal components account for more variable than one of the original variables, were retained. A loading of |0.4| was used to decide which variable was represented by each principal component (PC).

Ten components had an eigenvalue >1 and accounted for 88% of the total variance exhibited, but all the variables had a loading value < 0.4 in all the PCs. Thus, a PCA was not a useful approach for the dataset. The total variance explained by each of the five first components is shown in the table.

Table 1: Component matrix from PCA with 5 major components represented

|  | PC1 | PC2 | PC3 | PC4 | PC5 |
| --- | --- | --- | --- | --- | --- |
| Multiplication rate in goats | -0.23 | 0.21 | 0.10 | -0.09 | -0.20 |
| Multiplication rate in sheep | -0.27 | 0.14 | 0.08 | 0.11 | -0.18 |
| Production rate in sheep | -0.17 | 0.25 | -0.03 | 0.20 | 0.12 |
| Production rate in goats | 0.14 | 0.26 | -0.03 | -0.06 | 0.21 |
| Net production rate in sheep | 0.26 | -0.01 | -0.13 | 0.01 | 0.32 |
| Net production rate in goats | 0.20 | -0.07 | -0.15 | 0.07 | 0.43 |
| Mortality rate in goats | 0.21 | 0.02 | 0.21 | -0.01 | -0.19 |
| Mortality rate in sheep | 0.26 | -0.06 | 0.13 | 0.01 | -0.23 |
| Intake rate in goats | 0.19 | -0.11 | 0.06 | 0.11 | -0.33 |
| Intake rate in sheep | 0.16 | -0.18 | 0.09 | 0.06 | -0.33 |
| Offtake rate in goats | 0.28 | -0.12 | -0.09 | 0.10 | 0.19 |
| Offtake rate in sheep | 0.31 | -0.09 | -0.07 | 0.05 | 0.09 |
| Parturition rate in sheep | 0.15 | 0.31 | 0.05 | 0.31 | -0.07 |
| Parturition rate in goats | 0.17 | 0.31 | 0.18 | -0.19 | 0.11 |
| Prolificacy rate in goats | -0.16 | -0.17 | 0.18 | 0.40 | 0.15 |
| Prolificacy rate in sheep | 0.00 | -0.17 | 0.42 | -0.17 | 0.12 |
| Twin rate in sheep | 0.00 | -0.17 | 0.42 | -0.17 | 0.12 |
| Twin rate in goats | -0.16 | -0.18 | 0.18 | 0.39 | 0.14 |
| Triplet rate in goats | 0.04 | -0.11 | 0.12 | 0.08 | 0.13 |
| Fecundity rate in sheep | 0.15 | 0.29 | 0.13 | 0.29 | -0.05 |
| Fecundity rate in goats | 0.144 | 0.28 | 0.25 | -0.10 | 0.16 |
| Net fecundity rate in goats | 0.13 | 0.28 | 0.25 | -0.11 | 0.15 |
| Net fecundity rate in sheep | 0.14 | 0.29 | 0.13 | 0.30 | -0.06 |
| Net prolificacy rate in sheep | -0.06 | -0.16 | 0.40 | -0.15 | 0.10 |
| Net prolificacy rate in goats | -0.18 | -0.16 | 0.19 | 0.36 | 0.11 |
| Abortion rate in sheep | 0.15 | -0.04 | 0.13 | 0.01 | -0.04 |
| Abortion rate in goats | 0.01 | 0.11 | 0.11 | 0.08 | -0.01 |
| Stillbirth rate in sheep | 0.15 | -0.01 | 0.04 | -0.05 | 0.03 |
| Stillbirth rate in goats | 0.09 | -0.03 | -0.05 | 0.11 | 0.13 |
| Dystocia rate in sheep | 0.21 | -0.11 | 0.02 | 0.08 | -0.13 |
| Dystocia rate in goats | 0.23 | -0.06 | -0.02 | 0.14 | -0.09 |
| *Eigenvalues* | *7.96* | *4.60* | *3.28* | *2.57* | *2.12* |
| *% de variance* | *25.7* | *14.8* | *10.6* | *8.3* | *6.8* |
| *Cumulative (%)* | *25.7* | *40.5* | *51.1* | *59.4* | *66.2* |

**Annex 5: K-means clustering analysis results**

A cluster analysis using a K-means clustering approach was done with the following indicators: sheep net fecundity rate, goat net fecundity rate, sheep flock multiplication rate, goat flock multiplication rate, sheep net production rate and goat net production rate.

Clustering of this variables was investigated using the Euclidian distance. To determine the optimal number of clusters the Average Silhouette Method was used, the results showed that 2 clusters maximize the average silhouette values but, as it is shown in figure below, not clear delineations of the clusters were found, with overlapping in some areas.


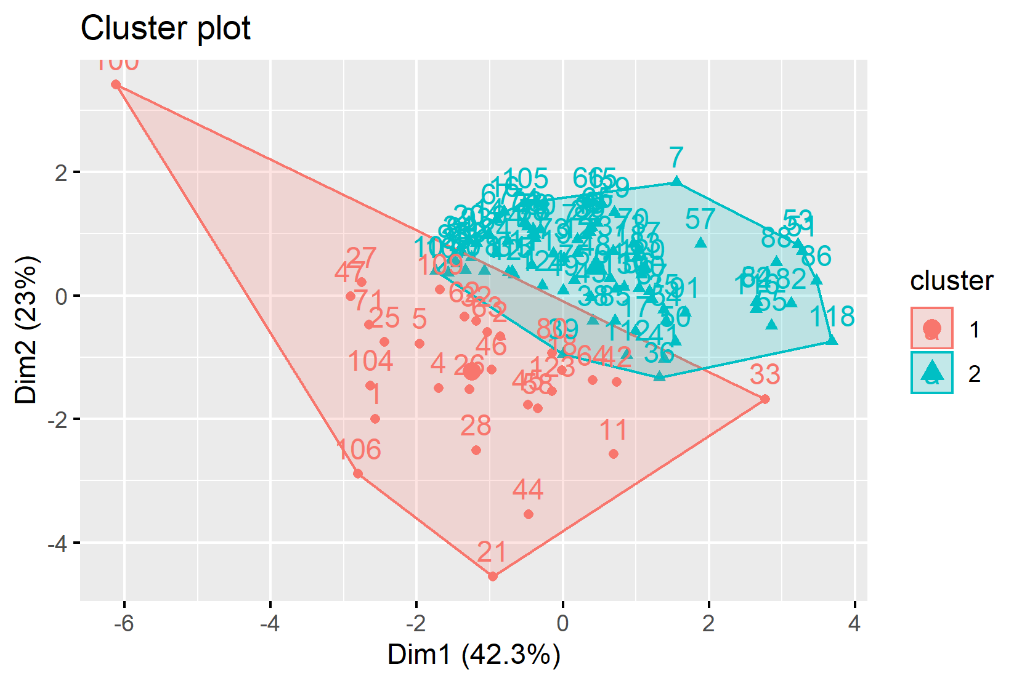

Supplement: Supplementary file 1 [file mmc1.docx]
